# Ten years of Gulf Coast ecosystem restoration projects since the *Deepwater Horizon* oil spill

**DOI:** 10.1073/pnas.2213639119

**Published:** 2022-09-16

**Authors:** Heida L. Diefenderfer, Larry D. McKinney, Walter R. Boynton, Kenneth L. Heck, Barbara A. Kleiss, Deepak R. Mishra, Holly Greening, Albert A. George, Bethany A. Carl Kraft, Catherine L. Kling

**Affiliations:** ^a^Coastal Sciences Division, Pacific Northwest National Laboratory, Sequim, WA 98382;; ^b^School of Environmental and Forest Sciences, University of Washington, Seattle, WA 98195;; ^c^Harte Research Institute for Gulf of Mexico Studies, Texas A&M University, Corpus Christi, TX 78412;; ^d^Chesapeake Biological Laboratory, University of Maryland, Solomons, MD 20688;; ^e^Dauphin Island Sea Lab, Dauphin Island, AL 36528;; ^f^School of Marine and Environmental Sciences, University of South Alabama, Mobile, AL 36688;; ^g^Department of River–Coastal Science and Engineering, Tulane University, New Orleans, LA 70118;; ^h^Department of Geography, University of Georgia, Athens, GA 30602;; ^i^CoastWise Partners, Bradenton, FL 34211;; ^j^South Carolina Aquarium, Charleston, SC 29401;; ^k^Volkert, Inc., Mobile, AL 36602;; ^l^Dyson School of Applied Economics and Management, Cornell University, Ithaca, NY 14850

In 2020, the National Academies of Sciences, Engineering, and Medicine (NASEM) Gulf Research Program created the Committee on Long-Term Environmental Trends in the Gulf of Mexico. Our committee was tasked to consider the synthesis of additive, synergistic, and antagonistic cumulative effects resulting from ecosystem restoration following the 2010 *Deepwater Horizon* (DWH) oil spill. This anticipated multidecadal restoration was made possible by dedicated settlement monies, distributed over the past decade as governed by the RESTORE Act of 2012 and other legal vehicles, which are today approaching one-half spent or committed. Thus, in our view, it is important to take stock of progress and, looking forward, to make recommendations regarding strategies for evaluation and management.

It is timely to examine the collective effects of coastal restoration across the five US states because the spatial and temporal coordination of restoration throughout a geographic region can substantially increase the return on investment (1). By the end of 2021, more than 570 environmental restoration projects were underway or completed. These included at least 152 focused on habitat restoration and enhancement, 82 on species restoration, and 47 on water-quality restoration and management, conducted by numerous state, federal, nonprofit, and other entities.

Synergistic and antagonistic interactions are a basic and cross-cutting concept addressed throughout our recent report (2). Ecological synergies occur when, for instance, living vegetated shoreline and oyster reef restorations interact to create seagrass meadows between them, or seagrass meadows and salt marshes interact to increase fish productivity (3). Conversely, coastal restoration utilizing freshwater and sediment diversions of river water could prove to have antagonistic effects. While mineral sediments may increase wetland volume and area, the changes in salinity, water quality, and substrate could be deleterious for marsh persistence, oysters, and other estuarine biota in the diversion area (4–6).

A robust consideration of restoration-related changes must also include the impacts of acute events (e.g., hurricanes) and long-term environmental trends (e.g., sea-level rise) on the valuable resources along the dynamic, spatially variable coastline of the US Gulf of Mexico (GoM). Yet, while the US West and East Coasts have hosted environmental synthesis centers since 1995 and 2011, respectively, the Gulf Coast does not have a scientific body performing similar functions. Here we argue that the time for synthesis of data and products from GoM restoration projects is now and the need is urgent.

One way to synthesize and evaluate large-scale restoration involves applying the concept of cumulative effects. The cumulative effects of restoration are the collective additive, synergistic, antagonistic, or null effects of all restoration activities that occur within a setting defined by common or connected characteristics of hydrology, geomorphology, ecology, ecological function, and biodiversity. There remain three critical challenges to the measurement of effects that must be addressed to encompass the full cumulative effects of large-scale restoration:

1) understanding and accounting for synergistic and antagonistic effects of restoration;
2) incorporating long-term trends and acute events in background environmental conditions; and
3) evaluating the spatial and temporal scale of restoration effects relative to other environmental changes.

In ecosystem restoration practice, inherent constraints make carefully designed experiments the exception, not the norm, and this tradition appears to have continued across the GoM since settlement-funded restoration began. The GoM itself or individual estuaries, bays, and watersheds are the experimental units for restoration effects and cannot be replicated to support a formal experimental design. Restoration since the spill has proceeded partially in a coordinated fashion but also has occurred in an ad hoc manner due to the manifold participants including five US states, multiple federal agencies, and two new organizations (NASEM’s Gulf Research Program and a federal agency, the Gulf Coast Ecosystem Restoration Council). Eighteen funding streams empowered by the settlements (appendix A of ref. 2) produce intersecting timelines of regulatory and environmental requirements.

In our report we reviewed the available data describing the nonstationary environmental trends facing the region. Against that backdrop, we then assessed approaches for evaluating the cumulative effects of restoration at multiple scales in the absence of an overarching, a priori study design by the restoration programs. Here we summarize the rationale for and essential features of an approach to synthesize the cumulative effects of restoration as recommended in our report, an approach rooted in lines of evidence and comparative analysis methods already proven on the Gulf Coast and elsewhere, which can and should be started now.

## Synergism and Antagonism in Ecosystem Restoration

Cumulative interactions—whether they are between drugs in the human body, or ecological interactions within an ecosystem, or among restoration projects—are generally considered as additive (the sum of their parts), synergistic (interactions are greater than the sum of the parts), antagonistic (interactions are less than the sum of the parts), or null in effect. Any of these interactions may be viewed as positive or negative, depending on the objectives, values, and perspectives of assessors. Understanding the potential end results of these interactions and how they can enhance restoration, both through positive synergies and by avoiding negative antagonistic interactions, should be a desirable goal for all restoration funders.

**Fig. 1.**
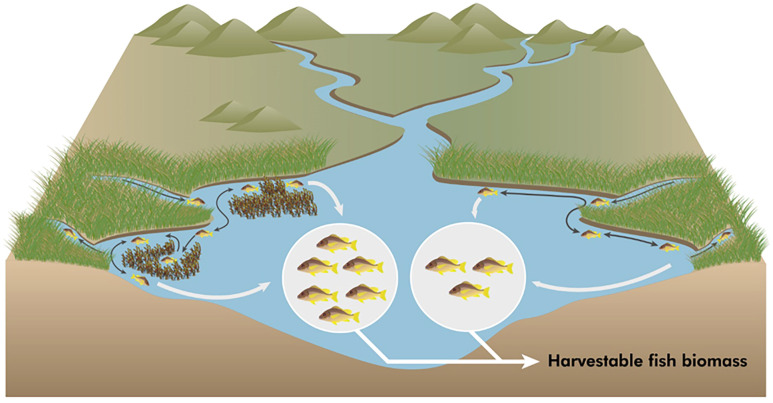
Example of synergism in coastal restoration. Fish move among complex habitats such as seagrass meadows and salt marshes in coastal seascapes (dark gray arrows). By restoring these habitats in close proximity to one another we might improve the habitat values, productivity, and carrying capacity of coastal seascapes for fish and fisheries (light gray arrows and ellipses). Symbols courtesy of the Integration and Application Network, https://ian.umces.edu/symbols. After figure 4 in ref. 3.

Although understanding remains incomplete and worthy of pursuit, some evidence exists for each of these types of interactions among and between human-constructed ecosystem restoration projects (Fig. 1). Ecosystem restoration may intentionally facilitate synergistic interactions among habitats (7) or species (8, 9).

Incomplete understanding poses a vulnerability for effective planning and engineering. Activities may blunt or, conversely, augment the expected gains from restoration. This has the potential to either reduce the cost effectiveness of restoration projects if unexpected antagonistic effects occur or to generate larger returns than expected if unexpected synergistic effects occur. Syntheses of cumulative effects focused on certain types of restoration method (e.g., living shorelines), or of two or more methods used in proximity to one another, therefore have the potential to directly inform advances in future ecosystem-restoration applications. A spatial example is that effects driven by localized environmental conditions that may not be replicated elsewhere may lead to a false indication of the value of restoration. A temporal example is when effects occur in the short run but disappear over longer durations (or vice versa, time lags that delay the appearance of restoration effects). The true expected outcomes of restoration may be confused with the activities implemented or with preliminary initial outputs (e.g., the number of acres treated with restoration measures).

## Acute Events and Long-Term Trends

Acute events and long-term environmental changes could have profound effects on the individual impacts and cumulative effects of restoration projects in the GoM. As well as inherent evolution of the system, anthropogenically exacerbated nonstationarity affects the physical and biological components of the ecosystem in wide-ranging ways (chapter 2 of ref. 2). Such changes have the potential to confound the detection of a measurable effect from even very large-scale and intensive restoration actions.

Climate-change impacts affecting restoration outcomes (10) include, for instance, tropicalization, the suite of changes as tropical species’ ranges expand into formerly temperate environments where changes in abundance, composition, and interactions in coastal plant communities and fauna can be significant (11). Other major ecosystem drivers that are changing include the magnitude and dynamics of freshwater, nutrient, and sediment inputs, with manifold impacts on coastal processes. High-resolution information describing the spatial pattern of physical and environmental systems is lacking; for example, sea-level rise relative to subsidence, despite its importance to plant community establishment and resilience, and wetland productivity via above- and below-ground carbon storage, despite being a key indicator of ecosystem function and effectiveness of restoration projects. Such trends and data gaps make it critical to note that types of restoration actions successful in each location in the past may not be successful in the future. Conversely, they may be more successful! This is the dilemma that profound uncertainty presents to restoration planners facing rapid change, amid the cumulative interacting effects of ecosystem modifications.

Given the variable but also substantial rate of change that is occurring within the GoM, there are imperatives to make long-term data collection and synthesis a top priority and to systematically include the latest data synthesis in planning for project implementation and adaptive management.

## Relative Scale of Restoration Effects

In the absence of sufficient scale of restoration activities, cumulative effects of restoration may not be measurable or may prove to be easily obscured by background variability. An example is the attempts to cure an annually occurring hypoxic zone in the GoM, which forms during the summer months due to excess nutrients mainly from agricultural activities in the Mississippi–Atchafalaya River Basin. The goal for reducing the size of the hypoxic zone, originally set in the 2001 Action Plan (12), is not currently being met and has not been met in any single 4-y period. Currently, using Iowa as an example, 3% of the state’s row crops are treated with suitable conservation actions, whereas the state estimates that 90% would need to be treated for nutrient reduction to reach the target needed to produce a measurable decrease in the size of the zone. Moreover, while the quantity of nitrogen delivered to the Gulf is an important predictor of the size of the hypoxic zone, spring river discharge rates as well as the presence of hurricanes and ocean currents can generate antagonistic or synergistic effects with large consequences for the size of the zone observed in any given year. It is evident from this example that nowhere near enough reduction of nitrogen loss has been made to expect detectable ecosystem responses in the GoM. To achieve desired benefit–cost ratios from investments in water quality and other post-DWH improvements on the GoM (13) it is important to appropriately scale expectations for the effects of restoration using suitable statistical tools (14).

Spatiotemporal scales of both restoration and background environmental conditions factor into the expression of unexpectedly high rates of ecosystem function, termed hot spots (places) or hot moments (periods) (HSHM) (15). Combined “spot–moments,” termed “ecosystem control points” by ref. 16, should be considered in restoration planning because of potential short-term, episodic, or localized activations. The ordinary monitoring of ecosystem restoration outcomes may overlook the effects of ecosystem processes in such places, which may not be trivial when considered cumulatively throughout the restoration trajectory. Incorporating HSHM and control points allows planners to tailor restoration to achieve beneficial cumulative effects and appropriately focus spatiotemporal aspects of monitoring strategies.

## Call for Synthesis and Evaluation of the Cumulative Effects of Large-Scale Restoration

Synthesis in ecology aims to discover new knowledge by bringing information together and has been defined for estuarine and coastal science as “the inferential process whereby new models are developed from analysis of multiple data sets to explain observed patterns across a range of time and space scales” (17). Why is post-DWH synthesis on the GoM important? Because it is the only way we can determine whether restoration efforts beyond the project scale, say at the regional or entire Gulf scale, have produced the results intended. This provides the opportunity to make midcourse corrections if they are needed. Why hasn’t synthesis happened? There are a number of reasons, but four important ones are that synthesis is difficult, synthesis takes time, the GoM is exceedingly complex by many measures, and in contrast to other US continental coasts (18–20) no regional entity has been charged with carrying out synthesis or stepped forward to do so.

Our review found that management of recoveries in Chesapeake, Tampa, and Galveston Bays, led by recognized regional entities, implemented essential elements of a lines-of-evidence approach to synthesis, though not using this terminology (2, 21–23). Restoration practitioners developed hypotheses regarding what was causing harm to ecosystem health. Data, analyses, and models from a wide range of scientific actors were marshalled and organized to address those hypotheses. Such organization falls into categories of lines of evidence (table 3.3 of ref. 2) that large-scale restoration programs on all continental US coasts have used, along with causal criteria for synthesis and evaluation (24, 25).

Using multiple lines of evidence helps to compensate for the inability to use traditional experimental designs, lack of reference conditions, lack of replication, difficulties in establishing causality, and often the shortage of appropriate data (25). Adoption of comparative cross-system analysis methods (26) for the 34 US GoM estuaries could provide an initial step bridging spatial gaps between existing estuary-scale assessments of cumulative effects and future GoM-wide assessments. Comparative analysis is one of five categories of coastal synthesis studies along with analysis of time series data, balance of cross-boundary fluxes, system-specific simulation modeling, and general systems simulation modeling (17). Critically, through the process of organizing results for hypothesis testing and modeling, gaps may be identified and prioritized for research. In this manner, knowledge is iteratively refined year to year, and the improved understanding of the system informs the selection of natural resource management actions in an adaptive management framework.

Synthesis and evaluation outcomes of interest to researchers, policymakers, or other stakeholders may be typical environmental and restoration endpoints such as ecosystem processes (e.g., sedimentation) or the type or biodiversity of restored systems (e.g., wetlands). Alternatively, they may be focused on ecosystem services from specific organisms (e.g., oysters) or performance targets (e.g., water quality). While they may also understandably be focused on political or community units (e.g., defined by state boundaries), the use of ecological boundaries for studies is encouraged, particularly where estuaries or watersheds are bistate. The use of ecological boundaries for analysis tends to produce greater fidelity and utility than environmental analyses conducted according to jurisdictional boundaries. Given all of these needs, the lack of an entity charged with conducting environmental synthesis across the GoM region is counterproductive to the cost-effective and impactful implementation of settlement monies through the restoration of productive ecosystems.
